# Effects of vitamin D in the elderly population: current status and perspectives

**DOI:** 10.1186/2049-3258-72-32

**Published:** 2014-09-28

**Authors:** Olivier Bruyère, Etienne Cavalier, Jean-Claude Souberbielle, Heike A Bischoff-Ferrari, Charlotte Beaudart, Fanny Buckinx, Jean-Yves Reginster, René Rizzoli

**Affiliations:** Department of Public Health, Epidemiology and Health Economics, University of Liège, CHU Sart-Tilman, Bât B23, Liège, 4000 Belgium; Department of Clinical Chemistry, University of Liège, CHU Sart-Tilman, Liege, Belgium; Department of Biology, Paris Descartes University, Necker Hospital, Paris, France; Geriatric Clinic, Zurich University Hospital and Center for Aging and Mobility, Zurich, Switzerland; Zurich University Hospital and Waid Hospital, Zurich, Switzerland; Department of Rehabilitation and Geriatrics, Geneva University Hospitals and Faculty of Medicine, Geneva, Switzerland

## Abstract

Besides its well-known effect on bone metabolism, recent researches suggest that vitamin D may also play a role in the muscular, immune, endocrine, and central nervous systems. Double-blind RCTs support vitamin D supplementation at a dose of 800 IU per day for the prevention of falls and fractures in the senior population. Ecological, case–control and cohort studies have suggested that high vitamin D levels were associated with a reduced risk of autoimmune diseases, type 2 diabetes, cardio-vascular diseases and cancer but large clinical trials are lacking today to provide solid evidence of a vitamin D benefit beyond bone health. At last, the optimal dose, route of administration, dosing interval and duration of vitamin D supplementation at a specific target dose beyond the prevention of vitamin D deficiency need to be further investigated.

## Background

The role of vitamin D in bone health has been known for over a century. More recent researches suggest that vitamin D may also play a role in the muscular, immune, endocrine, and central nervous systems. The objective of the current paper is to critically review observational and interventional studies on the potential effect of vitamin D on health outcomes among the elderly population. The vitamin D assays and the optimal vitamin D level are also discussed.

## Vitamin D assay and thresholds of vitamin D status

If all experts now agree that 25-hydroxy-vitamin D (25(OH)D) is the biomarker of choice to evaluate patients’ vitamin D status, the level of 25(OH)D that would be considered “normal” is more debated. Indeed, as the level of 25(OH)D fluctuates according to seasons, the reference ranges observed in “healthy” populations should be different in summer vs. winter, which does not make sense. Accordingly, all experts agree that a threshold defining vitamin D deficiency should be determined in relation to clinical outcomes, i.e. a value below which a detriment for health could be expected. This threshold is different whether we consider the general population or diseased patients. For the first ones, the Institute of Medicine recommends a target of 20 ng/mL and proposes Reference Dietary Intakes (RDI) that should help 97.5% of the population to reach this level [1]. These RDIs are of 400 IU from birth to 1 year old, 600 IU from 1 to 70 years old and 800 IU above 70 years. It should be noted, however, that other references intake values have been suggested based on other methodologies [2]. Anyway, in western populations, with a light sunshine exposure, no UVB synthesis from late fall to early spring and a diet containing limited amounts of vitamin D, a basic supplementation of 400–600 IU per day should thus be necessary to achieve these goals, at least in winter. It should be noted that this supplementation could be performed without preliminary 25(OH)D determination as the 20 ng/mL threshold is only a recommendation (no harm will happen if the subject presents a value slightly lower or higher than 20 ng/mL) and the doses proposed are totally safe.

For patients, and particularly for patients presenting kidney, bone or phosphocalcic disorders, many experts consider however that this 20 ng/mL threshold is too low [3]. They thus suggest a target of 30 ng/mL, according to different levels of proof, like the relation between parathormone (PTH) and vitamin D (even if the results from the studies show a substantial heterogeneity in this relationship), the prevalence of signs of mineralization defects below 30 ng/mL [4] or, most importantly, the levels reached by patients in treated group of randomized controlled trials showing a positive effect of vitamin D vs placebo (mainly studies on fracture or risk or fall prevention [5, 6]). In this context, there is some evidence that a benefit is expected if the patient’s 25(OH)D level is higher than the cut-off and a monitoring of 25(OH)D levels is thus mandatory. Recently, the European Society for Clinical and Economic Aspects of Osteoporosis and Osteoarthritis (ESCEO) recommended that 50 nmol/L (i.e. 20 ng/mL) should be the minimal serum 25-(OH)D concentration at the population level and in patients with osteoporosis to ensure optimal bone health [7]. However, ESCEO also states that in fragile elderly subjects who are at elevated risk for falls and fracture, a minimal serum 25-(OH)D level of 75 nmol/L (i.e. 30 ng/mL) should be reached for the greatest impact on fracture. The doses necessary to reach the target of 30 ng/mL are definitively higher than the ones necessary to obtain 20 ng/mL. They can reach 800–2000 IU per day or 24000–60000 IU per month, and require a control 3 months after initiation of the treatment, when a plateau is reached. According to the 25(OH)D levels reached, the doses can be tailored to maintain the patient in the 30–50 ng/mL range. This range is totally safe as it is naturally obtained in populations exposed during all year to high UV radiation, like the Maasaï, who present a mean 25(OH)D concentration of 46 ng/mL with extreme values ranging from 25 to 75 ng/mL [8]. Compliance of the patient with the treatment is however problematic and yearly controls should be performed. Daily, weekly or monthly doses are equivalent in rising and maintaining 25(OH)D levels and patients should choose which form they prefer. Large, yearly doses, should however be abandoned as totally non physiologic, and even potentially harmful [9].

In the nineties and early twenties, most laboratories were using the DiaSorin RIA to assess 25(OH)D levels. The cut-offs of 20 and 30 ng/mL are notably derived from studies that were using this assay device. However, the increasing number of requests has led most of the clinical laboratories to switch to methods presenting a larger throughput, *i.e*. automated immunoassays or liquid chromatographs coupled with two mass spectrometers in tandem (LC-MS/MS). The determination of 25(OH)D concentration is however far from an easy task and several important problems, among which the very high lipophilic nature of the molecule and its strong association with its carriers, vitamin D binding protein (VDBP) and albumin have to be overcome to correctly assess the parameter [10]. VDBP can be present at different concentrations depending on some physiological or pathological conditions, like race [11], pregnancy or chronic kidney disease, which could influence the kinetic of the liberation of the molecule [12, 13]. Vitamin D can be found as vitamin D2 or D3 and the assay should measure both 25(OH)D2 and 25(OH)D3 [14]. Different other metabolites of vitamin D can be present in the serum of the patients at different levels, possibly interfering with either immunoassays or LC-MS/MS methods [15]. Just like any other immunoassays, vitamin D assays are prone to heterophilic antibodies interference, leading to potential spurious results [16]. Last but not least, the lack of standardization of the different assays remains a major problem. A worldwide standardization program (Vitamin D Standardization Program, VDSP), coordinated by the Centers for Disease Control and Prevention (CDC), the National Institute of Standards and Technology (NIST) and the University of Ghent, Belgium, is ongoing to improve the standardization and will certainly reduce the variation observed between methods and laboratories in healthy individuals. Nevertheless, different problems will remain in special populations, like pregnant women or hemodialyzed patients, for whom standardization seems to be less efficient [12, 13]. Moreover, neither 25(OH)D2 standardization nor 25(OH)D2 recovery will be solved by the VDSP. Finally, re-standardization will impact the traditional “20” or “30” ng/mL values that are used as clinical cut-offs to define vitamin D sufficiency. Indeed, as already mentioned, these cut-offs derive from studies that generally used the DiaSorin RIA for 25(OH)D measurements. Using these cut-offs with immunoassays or LC-MS/MS methods that are differently calibrated is thus hazardous. Re-standardization will reduce method-to-method variations, but will consequently also impact the cut-off values, that will need to be updated according to the new standard.

## Effects of vitamin D on falls

In a meta-analysis of 8 double-blind RCTs including a total of 2426 individuals aged 65 and older [6], anti-fall benefits of vitamin D supplementation were observed from a dose of 700 IU per day onwards. In a re-analysis requested by the Institute of Medicine [17], when treatment was the only predictor (regardless of dose level), there was a significant reduction in the odds of falling: OR = 0.73 [0.62, 0.87]; p = .0004. When the model was expanded to capture the impact of both high dose and low dose treatments, high dose vitamin D treatments (700 to 1000 IU vitamin D per day) reduced the odds of falling (OR = 0.66 [0.53, 0.82]; p = .0002), while low dose vitamin D treatments did not (OR = 1.14 [0.69, 1.87]; p = .61). In the Report on Vitamin D (FCN Report (2012), there was a 38% reduction in the risk of falling with a treatment duration of 2 to 5 months and a sustained significant effect of 17% fall reduction with a treatment duration of 12 to 36 months with vitamin D supplements/doses of 700 to 1000 IU. Thus, benefits of vitamin D supplementation of 700 to 1000 IU per day on fall prevention are rapid and sustained and concern all subgroups of the senior population [6].

## Effects of vitamin D on bone

### Results from double-blind randomized controlled trials

Vitamin D is essential for bone growth [18, 19] and bone health preservation [20]. Higher 25(OH)D levels are associated with higher bone density in younger and older adults [21]. Also, in various double-blind RCTs, vitamin D supplementation increased bone density and reduced bone loss [22, 23]. In a meta-analysis summarizing the evidence of 12 double-blind RCTs involving 42279 individuals aged 65 and older, oral vitamin D supplementation reduced the risk of hip fracture by 18% and the risk of any non-vertebral fracture by 20% [5]. However, similarly to fall prevention, the benefit on fracture prevention depends on the dose of vitamin D. Fracture prevention required a received dose (treatment dose*adherence) of more than 482 IU vitamin D per day. The primary use of received dose (dose*adherence) as opposed to treatment dose from double-blind RCTs allowed for the assessment of anti-fracture efficacy by a dose that accounts for the low adherence in several recent large trials [24, 25]. Any lower received dose than 482 IU per day did not reduce fracture risk at either any non-vertebral site or the hip. Similarly to the data on fall prevention with vitamin D, at the highest received dose of vitamin D (>482 IU per day) the prevention of non-vertebral fractures was present in all subgroups of the older population independently of age and type of dwelling [5]. Notably, there was a suggestion that vitamin D3 was superior to vitamin D2 for both fall and fracture prevention [5, 6].

A participant level meta-analysis from 11 double-blind RCTs (31022 individuals with mean age 76 years, 91% women sustaining 1111 incident hip and 3770 non-vertebral fractures) assessed the effect of actual dose of vitamin D on fracture reduction. Actual dose considered adherence to treatment and additional vitamin D intake outside the study medication. In this pooled analysis, fracture reduction was only present at the highest actual intake of 800 IU of vitamin D per day (range: 792 to 2000 IU/d) with a 30% reduction at the hip and 14% reduction at any non-vertebral site independently of vitamin D treatment, age group, gender, type of dwelling and study [26]. This study further suggested that the typical intent-to-treat results for vitamin D, which was replicated by the authors with a non-significant 10% reduction at the hip and 7% reduction at any non-vertebral site, may underestimate the benefit of vitamin D supplementation and explain the conflicting results of other meta-analyses [26].

### Results from meta-analyses on fracture prevention that also included open-design trials

A review and meta-analysis commissioned by the US Department of Health and Human Services (HHS) has addressed the effect of vitamin D supplementation on all fractures in postmenopausal women and men aged 50 and older [27]. The pooled results for all fractures included 10 double-blinded and 3 open design trials (n = 58712). However, it did not support a significant reduction of fractures with vitamin D (pooled odds ratio = 0.90; 95% CI 0.81-1.02). The report suggested that the benefit of vitamin D may depend on additional calcium and may be primarily seen in institutionalized individuals, which is consistent with the meta-analysis of Boonen et al. [28].

The DIPART group conducted a patient-based meta-analysis including 7 large trials on vitamin D with 68500 individuals aged 47 and older [29] : two open design trials [30, 31], one trial with intra-muscular vitamin D, and 4 of the 10 double-blind RCTs included in the 2009 meta-analysis described above (one RCT using intermittent vitamin D2 doses without calcium [32], one RCT with 400 IU of vitamin D3 without calcium [33], one trial with 800 IU of vitamin D3 per day with and without calcium and less than 50% adherence [25], and one trial with 400 IU of vitamin D with calcium [24]). On the basis of the inclusion criteria, a reduced overall risk of fracture (hazard ratio = 0.92; 95% CI 0.86 to 0.99) and a non-significant reduction of hip fractures (hazard ratio = 0.84; 95% CI 0.70 to 1.01) was found for trials that used vitamin D plus calcium. Vitamin D supplementation alone, irrespective of dose, did not reduce fracture risk. It was concluded that vitamin D, even in a dose of 400 IU of vitamin D per day reduces the risk of fracture if combined with calcium. Notably, this regimen was tested in 36282 postmenopausal women in the Women’s Health Initiative Trial over a treatment period of 7 years and did not reduce the risk of fracture [24].

A most recent 2014 trial-level meta-analysis (76497 participants) was based on a mix of trials with blinded and open designs, follow-up periods that were as short as 1 month, administered doses and compliance that ranged widely, and endpoints that ranged from primary to secondary along with un-pre-specified, and consequently were adjudicated and non-adjudicated. However, despite the great variety and mixed quality of trials, the authors documented a significant 8% reduction for total fractures and a significant 16% reduction for hip fractures for vitamin D plus calcium supplementation [34].

### Discussion on the meta-analyses that also included open-design trials

In all 3 meta-analyses reviewed above, dose heterogeneity may have been missed due to the inclusion of open design trials plus a dose evaluation that did not incorporate adherence to treatment. A dose–response relationship between vitamin D supplementation and fracture reduction as documented for the two 2009 meta-analyses of double-blind RCTs [5, 6], is supported by epidemiologic data showing a significant positive trend between serum 25(OH)D concentrations and hip bone density [21] and lower extremity strength [35, 36]. Factors that may obscure the benefit of vitamin D supplementation are low adherence to treatment [25], too low doses of vitamin D, or the use of the less potent vitamin D2 [37, 38]. Furthermore, open design trials [30] may bias results towards the null because vitamin D is available over the counter.

### Conclusion on falls and fractures

Based on evidence from RCTs, oral vitamin D supplementation reduces both falls and non-vertebral fractures, including those at the hip. However, these benefits are dose-dependent and a dose of 700–1000 IU of vitamin D per day is required to assure both fall and fracture prevention in older adults.

## Effects of vitamin D on muscle

Proximal muscle weakness is a prominent feature of the clinical syndrome of vitamin D deficiency [39]. Muscle manifestations such as proximal muscle weakness, diffuse muscle pain and gait impairments are well-known clinical symptoms of vitamin D deficiency [40]. The activation of vitamin D receptors (VDRs), which is expressed in human muscle tissue [41, 42] appears to stimulate protein synthesis in muscle [43]. Smaller and variable muscle fibres and persistence of immature muscle gene expression during adult life are found in mice lacking VDR [44]. These abnormalities persist after correction of systemic calcium metabolism by a rescue diet, whereas the bone phenotype is normalized after correction of calcium and phosphate plasma concentrations [45].

Most observational studies show a positive association between higher 25(OH)D status and better lower extremity function in older adults, a lower risk of functional decline [35, 46], a lower risk of future falls and a lower risk of nursing care admission [47], including two population-based studies from the US [36] and Europe [35].

Consistently, in several trials of older individuals at risk for vitamin D deficiency, vitamin D supplementation improved strength, function, and balance [48–50]. Most importantly, these benefits translated in a reduction in falls in some of the same trials [48–50]. In three recent double-blind RCTs supplementation with 800 IU vitamin D3 resulted in a 4-11% gain in lower extremity strength or function [48, 50], and an up to 28% improvement in body sway [48, 49] in older adults aged 65 and older within 2 to 12 months of treatment. Extending to trials among individuals with a lower risk of vitamin D deficiency and including open design trials, a recent meta-analysis by Stockton identified 17 RCTs that tested any form of vitamin D treatment and documented a muscle strength related endpoint. The authors suggested that based on their pooled findings, vitamin D may not improve grip strength, but a benefit of vitamin D treatment on lower extremity strength could not be excluded (p = 0.07) among individuals with 25(OH)D starting levels of > 25 nmol/l and the authors report a significant benefit among two studies with participants that started with 25(OH)D levels < 25 nmol/l [51]. In a more recent meta-analysis of Muir and Montero-Odasso, 13 randomized controlled trials were identified in seniors aged 60 years and older. In the pooled analysis, vitamin D supplementation had a significant benefit on postural sway and lower extremity mobility measured with the timed up and go and lower extremity strength [52].

Mechanistically, it has been suggested that 1,25-dihydroxyvitamin D binds to the nuclear VDR in muscle resulting in de novo protein synthesis [53, 54]. At a clinical level, this is supported by findings of three small trials in older adults, which documented an increase in type II muscle fibres after treatment with 1-alpha-calcidiol [43] or vitamin D2 [55] or vitamin D3 [56].

Consequently, evidence supports the use of vitamin D supplementation to improve muscle strength and function but additional studies may be needed to define the optimal treatment dose.

## Other potential effects of vitamin D in the elderly population

Many tissues without any obvious relationship with the calcium/phosphorus and/or bone metabolism are able to express the VDR, 1-alpha-hydroxylase, and 24-hydroxylase molecules. 25(OH)D enters these tissues and is locally hydroxylated into calcitriol which binds to the VDRs present in these cells. This “peripheral” production of calcitriol is not regulated by calciotropic hormones (PTH, FGF23, …), but seems dependent on the 25(OH)D concentration in the extra-cellular fluid of these tissues. This is the basis for the “non-classical” genomic effects of vitamin D that could be considered as “intracrine” by contrast with the classical endocrine effects of calcitriol. We also know that plasma calcitriol can exert rapid non genomic effects in some tissues such as muscle fibres or pancreatic beta-cells where it binds to membrane proteins resembling the VDR [57].

In addition to its effects on calcium/phosphorus metabolism, non vertebral fractures and falls, vitamin D may exert various other effects as suggested by numerous observational studies that reported positive associations between vitamin D deficiency (i.e. low circulating levels of 25(OH)D) and an increased risk for many diseases that remained significant after adjustment for confounders. Among these potential non classical effects, some may be highly relevant to the elderly.

- Vitamin D deficiency is associated with an increased risk for different cancers, especially colorectal [58] and breast [59].
- Globally, many experimental studies support the suppression of acquired immunity and the stimulation of innate immunity by vitamin D. VDRs and 1-alpha hydroxylase are present in T and B lymphocytes, macrophages and antigen-presenting cells. Calcitriol reduces the proliferation of the T-lymphocytes, especially T-helper 1 (Th1) and Th17 lymphocytes and the production of certain cytokines with inflammatory properties. On the other hand calcitriol stimulates the production of other cytokines with anti-inflammatory actions such as IL10 and favours Th2 and regulatory T lymphocytes phenotypes. This modulation of acquired immunity is believed to be beneficial in a number of auto-immune diseases as suggested by studies showing that vitamin D deficiency is associated with higher incidence and poorer outcomes of some auto-immune diseases [60], and to have global anti-inflammatory effects [61] that could be of help in many diseases as an adjunct to usual therapy [62]. As regards innate immunity, it is now known that macrophages or monocytes exposed to an infectious agent such as bacillus tuberculosis overexpress Toll-like receptors, VDRs, and 1-alpha hydroxylase. Provided that the 25(OH)D concentration in the cell’s extracellular liquid is sufficient, they produce 1,25(OH)2D which binds to the VDRs inducing the production of antimicrobial peptides such as cathelicidin which contributes to the destruction of the infectious agent [63]. This mechanism may explain partly the relationship between the frequency of some infectious diseases and low 25(OH)D concentrations found in epidemiological studies [64].
- Vitamin D deficiency has not only been found to be associated with an increased risk of major cardio-vascular events but also with cardio-vascular mortality in several studies [65]. Potential mechanisms are complex and involve both direct effects of vitamin D on vascular endothelial cells, and indirect effects through the control of the renin-angiotensin system and thus blood pressure, on the PTH secretion, insulin secretion and sensitivity, and inflammation [66].
- In non-dialyzed patients with chronic kidney disease, vitamin D deficiency is associated with albuminuria and a more rapid deterioration of renal function [67].
- Lower serum vitamin D concentrations are found in patients with Alzheimer’s disease compared to matched controls [68], and predict executive dysfunction in community-dwellers [69].
- Finally, vitamin D sufficiency is associated with a delayed mortality not only in prospective observational studies [70], but also in interventional studies, especially when associated with calcium [71].

These potential “non-classical” effects of vitamin D seem so impressive that a discussion on the level of evidence supporting them is necessary. Indeed, “association” does not mean “causality”, and it must be recognized that the effects mentioned above in the previous paragraph are mostly documented by observational (often prospective however) and experimental (cell culture, animal models…) studies. One important question is to know whether vitamin D supplementation is able to improve all or part of the disease/anomalies associated with vitamin D deficiency or whether the above-mentioned associations only reflect a poor health status. Several RCTs showing a better effect of vitamin D supplementation compared to placebo on these diseases or their complications exist to-date (see for example [72–79]). The results of these positive RCTs are however generally not applicable to the general population as they were targeted to specific groups [76–78], or were the results of secondary objectives of studies that had been designed to study another function [72], or concerned intermediate parameters and not “hard” (clinical) end-points [73–75, 79]. Furthermore, numerous RCTs have been “null” in that they showed no benefit, but also no disadvantage compared to placebo. To our knowledge, only two studies on the risk of fracture in elderly subjects that used very large vitamin D doses administered at very large intervals were “negative” (i.e. worse results in the vitamin D groups than in the placebo groups [9, 80]). Reasons that may explain the discrepancies between the results of these various studies are several. Among the most frequently cited are the use of vitamin D doses that are too low to expect any effect, a poor observance, and inclusion of subjects who were not vitamin D deficient. It must be acknowledged that when the RCTs that have tested the non classical effects of vitamin D (i.e. effects other than those on fractures, falls, and improvement of the calcium/phosphorus metabolism) are grouped in meta-analyses and evaluated according to an intent-to-treat analysis, no (or very minimal at best) effects of vitamin D could be ascertained [81]. Intent-to-treat analysis, which is necessary for a relevant evaluation of drugs according to the “Evidence-based-Medicine” concept, should however not be systematically applied to the evaluation of vitamin D effects which is not a drug *stricto sensu* (as well as to any other nutriment), or should be at least adapted. Having said that, we know that RCTs will remain the gold standard to definitely conclude that the above-discussed non classical effects of vitamin D are a reality. It seems thus important to define the conditions that will allow the best interpretation of the data (see Table 1 for a tentative suggestion of a list).

**Table 1:** **Parameters and conditions that should be controlled for an optimal evaluation of the effects of vitamin D in future RCTs**

Conditions	Actions
Conditions allowing an optimisation of the statistical power of the study (common conditions for trials of drugs and nutrients)	Sample size (number of participants) and trial duration must be appropriately calculated according to the frequency of the studied event in the recruited population. These points depend on the basal clinical status of the patients (larger sample and/or longer duration if the studied disease is not very active in the recruited patients)
	Adherence/observance must be optimized (for example, new technologies such as SMS that are sent the day just before the treatment must be taken, in case of intermittent dosage, allow an easy reminding for the patients)
Conditions specific to a vitamin D trial	Choose to administrate vitamin D3 instead of D2, specially in case of intermittent dosage
	Ensure that dietary calcium intakes of the participants are sufficient
	Treat with daily doses or, in case of intermittent dosage, choose doses that are not too high (<or = 100000 IU) and not too spaced out (ideally < or = 1 month)
	Choosing the dose will depend on the disease to be studied (search in the literature) but must be above 800 IU/day (often more)
	Possible vitamin D supplements that were taken by the patients before the study must be stopped (paradoxically, it was found in some studies with a poor observance that some patients in the placebo group received in fact more vitamin D during the trial than some patients in the vitamin D group)
	It will be important to recruit patients with low 25OHD serum levels (or at least much lower than the 25OHD levels that are targeted in the study) so that a frank increase of the 25OHD concentration may be observed on the one hand, and so that the placebo group is really insufficient/deficient on the other hand.

## Discussion

Three elements deserve further discussion.

### Dosing intervals

In 2010, a large double-blind RCT by Sanders et al., included 2256 community-dwelling women aged 70 years and older, to test the benefit of 500000 IU of vitamin D3 given orally once a year, on fall and fracture prevention [9]. In those women, mean age 76, considered to be at risk of fracture, 500000 IU of vitamin D once a year did not reduce, but instead increased the risk of falls by 15% and the risk of fractures by 26% compared to placebo, with the greatest increase in falls occurring during the first 3 months after dosing. These findings are consistent with another trial that tested 300000 IU of vitamin D2 as an intra-muscular injection once a year [80]. The temporal pattern of events may suggest that the high dose of vitamin D may have induced a “protective” reaction resulting in an acute decrease in 1,25-dihydroxyvitamin D [82]. Alternatively, the undocumented potential effect of vitamin D on muscle strength [36] and overall health (i.e. less infections and less hospital admissions [83]) in the Sanders trial, may have been an improvement in mobility which has ironically, led to increase opportunities to fall and fracture. As a result of the Sanders trial and given that the half-life of vitamin D is 3 to 6 weeks, a daily, weekly, or monthly dosing interval may be more advantageous and safer [84, 85].

### How to promote optimal vitamin D status

Studies suggest that supplementation of 700 to 1000 IU of vitamin D per day may bring the concentration in 25(OH)D of 50% of younger and older adults up to 75–100 nmol/L [86–88] Individuals with a lower starting level may need a higher dose of vitamin D to achieve desirable levels, while relatively lower doses may be sufficient in individuals who start at higher baseline levels. Due to seasonal fluctuations of 25(OH)D levels [89], some individuals may be in the desirable range during summer months, but their levels will not sustain during the winter months, even in sunny latitudes [90, 91]. Furthermore, several studies suggest that many older persons will not achieve optimal serum 25(OH)D levels during summer months suggesting that, among this population, vitamin D supplementation should be independent of season [91–93]. Even among younger persons, the use of sunscreen or sun-protective clothing may prevent a significant production of 25(OH)D [93]. Most vulnerable to low vitamin D levels are older individuals [91, 94], individuals living in northern latitudes with prolonged winters [89, 95], obese individuals [96], and individuals of all ages with dark skin pigmentation living in northern latitudes [21, 97, 98]. Naturally high 25(OH)D levels observed in healthy outdoor workers are 135 nmol/L [99] in farmers and 163 nmol/L [100] in lifeguards. As a first sign of toxicity, only serum 25(OH)D levels of above 220 nmol/L have been associated with hypercalcemia [101, 102].

### Reverse causality and confounding factors in observational studies

When analysing results of observational studies, it is important to consider potential reverse causality or residual confounding factors. For example, lifestyle factors, not always adequately recorded in observational studies, could influence circulating levels of vitamin D and, as such, could confound the association between 25(OH)D levels and incidence of diseases. On the other hand, 25(OH)D levels might not be responsible for the changes in outcomes of the diseases but disability in itself might influence the vitamin D status of the individual. In other words, serum 25(OH)D levels could just be a biomarker of severity of the diseases. In principle, these issues with reverse causality and confounding factors could be ruled out with RCTs.

## Conclusion

Results from ecological, case–control and cohort studies have shown that high vitamin D levels were associated with a reduced risk of bone fracture, falls, autoimmune diseases, type 2 diabetes, cardio-vascular diseases and cancer. Since the prevalence of vitamin D inadequacy is high, supplementation with vitamin D has then been recommended, especially in high risk and elderly population. Notably, evidence from double-blind RCTs support vitamin D supplementation at a dose of 800 IU per day for the prevention of falls and fractures in the senior population. Further, several studies reviewed in this paper suggest a potential effect of vitamin D in human health but large clinical trials are lacking today to provide solid evidence of a vitamin D benefit beyond bone health at all ages and fall prevention in the senior population. Additionally, the optimal dose, route of administration, dosing interval and duration of vitamin D supplementation at a specific target dose beyond the prevention of vitamin D deficiency needs to be further investigated. It is possible that the optimal level of vitamin D should be individualized, based on clinical and demographic characteristics of the subject and outcome.
